# The addition of *jogi*, *Micropogonias undulates*, affects amino acid content in kimchi fermentation

**DOI:** 10.1371/journal.pone.0300249

**Published:** 2024-04-04

**Authors:** Junghyun Park, Sojeong Heo, Gawon Lee, Tao Kim, Seung-Eun Oh, Mi-Sun Kwak, Do-Won Jeong

**Affiliations:** 1 Department of Food and Nutrition, Dongduk Women’s University, Seoul, Republic of Korea; 2 KookminBio Corporation, Seoul, Republic of Korea; Universidad Autonoma de Chihuahua, MEXICO

## Abstract

The effects of *jogi* (the fish Atlantic croaker, *Micropogonias undulatus*) on the production of physicochemical components, such as color, organic acids, and amino acids, in kimchi, a traditional fermented vegetable food of Korea, were determined. As fermentation progressed, the color change of *jogi*-added kimchi increased, but in comparison with that of the control group without *jogi*-added kimchi, was difficult to distinguish with the naked eye. Reducing sugar decreased in all experimental groups, and as fermentation progressed, kimchi with *jogi* showed a lower value. Acetic acid, citric acid, lactic acid, and ethanol, were highly produced in both types of kimchi, and above all, the *jogi—baechu—kimchi* group showed higher acetic acid and lactic acid contents than the control group. The increase and decrease of amino acids were similar in both types of kimchi. However, significantly, immediately after manufacture, the savory components aspartic acid and glutamic acid were detected higher than the control group. Subsequently, the fermentation tended to decrease as it progressed, but the content was higher than that of the control group. The above results show that *jogi* addition has a greater effect on the contents of amino acid, especially the savory component, than on the physicochemical components.

## Introduction

Kimchi is a representative lactic acid fermented vegetable food of Korea. The main ingredient of kimchi is *baechu* (kimchi cabbage, *Brassica rapa*), red pepper powder, garlic, green onions, ginger, and various salted seafood (*jeotgal*) are added to salted *baechu*, and fermented for a certain period of time [1, 2]. Kimchi combines the various flavors of organic acids and free amino acids and ingredients produced during fermentation to give it a unique taste. Among the constituents, free amino acids affect the quality of kimchi by affecting the growth of lactic acid bacteria, as well as taste [3]. The content of the free amino acids during fermentation is affected by the kind, amount, combination of the raw material, aging temperature, and more [4, 5].

Among the auxiliary ingredients added to kimchi, seafood is added in the coastal region to enhance the taste of kimchi and supplement the nutrients, such as amino acids [6–8]. Seafood is decomposed by enzymes that are autolytic, and during kimchi fermentation, produces kimchi microorganisms, giving off a savory taste and becoming a nutrient source for lactic acid bacteria [9]. Seafood added as minor ingredients for kimchi studied so far include sea squirt (*Styela clava*), *kwamegi* (semi-dried *Clupea pallassii* or *Cololabis saira*), dried pollack (*Gadus chalcogrammus*) powder, octopus (*Enteroctopus dofleini*), squid (*Decapodiformes*), hairtail (*Trichiurus lepturus*), and croaker (*Micropogonias undulatus*), as well as other seafood [6–8, 10–14].

The *jogi* (yellow croaker, *Micropogonias undulatus*) is a species of fish in the family Sciaenidae, which occurs around Korea, China, and Japan. The *jogi* contains 76.3 g of moisture, 19.0 g of protein, 4.0 g of lipids, 1.3 g of ash, and small amounts of minerals, such as sodium, potassium, calcium, and phosphorus, and vitamins, such as vitamin A, thiamine, riboflavin, and niacin per 100 g of edible part (The Korean Food Composition Table; http://koreanfood.rda.go.kr/eng/fctFoodSrchEng/main). As such, *jogi* is a food ingredient that is rich in protein.

In a previous study, we identified the effect of adding *jogi* to kimchi on microbial communities [14]. Contrary to the expectation that microbial species that break down proteins will predominate due to the high protein content of the *jogi*, the addition of *jogi* did not significantly affect the change in bacterial communities. Therefore, this study examined the physicochemical properties and the effect on the quality of kimchi according to the fermentation period while storing *jogi*-added kimchi, and sought to determine the correlation between the amino acid content produced during aging and the microbial bacteria.

## Materials and methods

### Kimchi

Kimchi, which was used for microbial community analysis [14], was used in this study. In brief, *baechu-kimchi* was home-made kimchi in Sangju, Gyeongsangbuk-do, and which was used as a control group. *Baechu-kimchi* was made by adding carrot, chives, garlic, ginger, *jeotgal*, leaf mustard, and red pepper into salted *baechu* and *jogi—baechu—kimchi* with approximately 5% *jogi* added to the total volume of the control group was used as an experimental group. Kimchi was immediately stored at 10 °C for 20 days, sampled every 5 days, and stored in a deep freezer at −80 °C. In order to reduce the prejudice of kimchi collected part, all samples were ground and used in the experiment.

### Color analysis

Kimchi sample for color difference analysis was ground with a blender, and 1 g was taken to measure the values of lightness (L), redness (*a*), and yellowness (*b*), which are the components of the Hunter color system, and Δ*E* values were derived. The chromaticity was measured by colorimeter (Chroma Meter CR-400, Konica Minolta, Tokyo, Japan) calibrated with a standard white plate (L = 93.28, *a* = −0.58, and *b* = 3.50). All experiments were conducted in triplicate.

### Reduction sugar analysis

For measurement of the reducing sugar, kimchi sample was homogenized, filtered through sterilized cheesecloth, and a filtrate was used. Reduction sugars were measured by a colorimetric method using dinitrosalicylic acid (DNS) [3, 15]. The DNS reagent of 3 mL was added to 1 mL of a 50-fold diluted filtrate, stirred well, reacted in boiling water for 5 min, and cooled to measure the absorbance at 550 nm by UV/VIS spectrophotometry (Optizen POP, Mecasys Co., Ltd, Daejeon, Republic of Korea), and this measurement was then converted into glucose. All experiments were conducted in triplicate.

### Organic acid analysis

Ground samples (5 g) were mixed with 45 mL of sterilized water. The mixture was sonicated for 10 min, and then filtered through a 0.2 μm syringe filter (Sartorius Stedim Biotech GmbH, Göttingen, Germany). The filtrate was analyzed by a custom service provided by the National Instrumentation Center for Environmental Management in Korea (http://nicem.snu.ac.kr/). The analyses were performed using the Dionex Ultimate 3000 HPLC system (Thermo Scientific, Waltham, MA, USA). Chromatographic separation was conducted with an Aminex 87H column (300 mm × 10 mm; Bio-Rad, Hercules, CA, USA). Gradient elution was carried out with 0.01N H_2_SO_4_. Injection volumes were 10 μL, and the column temperature and flow rate were 40 °C and 0.5 mL/min, respectively. The detection was performed using a refractive index (RI) detector (RefractoMAX520, ERC Inc., Saitama, Japan) at 210 nm. The organic acid contents of two samples prepared in the same conditions were analyzed in triplicate.

### Analysis of free amino acids

The same filtrate that was used for the analysis of organic acids was analyzed using a JASCO LC-2000 Plus Series HPLC system (JASCO, Tokyo, Japan). Chromatographic separation was conducted with an AccQ-Tag Amino Acids C18 Column (3.9 mm × 150 mm, 4 μm; Waters Corporation, Milford, MA, USA). Gradient elution was carried out with AccQ-Tag/water (solvent A; 10:90, v/v) and acetonitrile/water (solvent B; 60:40, v/v). The following binary mobile phase linear gradients were used: 100% A at 0 min, 98% A at 5 min, 93% A at 15 min, 90% A at 19 min, 67% A at 32–33 min, 0% A at 34–37 min, and 100% A at 38 min. Injection volumes were 10 μL, and the column temperature and flow rate were 37 °C and 1 mL/min, respectively. The detection was performed using a fluorescent detector. AccQ-Fluor Reagent Kit (WAT052880, Waters Corporation, Milford, MA, USA) was simultaneously used as derivatizing agents, according to the manufacturer’s instructions. The photodiode array (PDA) detector (MD-2018 Plus, JASCO, Tokyo, Japan) wavelength was set to 254 nm for the determination of the AccQ-Fluor Reagent-derivatized amino acids. The concentrations of individual free amino acids were determined using five-point calibration curves of the amino acid standard (WAT088122, Waters Corporation, Milford, MA, USA). The free amino acid contents of two samples prepared in the same conditions were analyzed twice.

### Biographical correlation analysis between bacterial communities and free amino acids

Bacterial communities at species level in the two types of kimchi were analyzed for correlation with the free amino acids using the Pearson correlation coefficient. Correlation analysis was performed using the Paleontological Statistics software package (PAST) version 4.13 [16].

### Comparative genomics

Comparative genomic analysis was performed to explain the production of amino acids produced in kimchi via genomic insights. Ten species assumed to be correlated with amino acid production were selected. Genome information was prioritized for species registered with complete genomes and type strain, and next selected as species registered with complete genomes, except for type strain and draft genomes. The genomic information for the 10 species was retrieved from the National Center for Biotechnology Information database (NCBI; http://ncbi.nlm.nih.gov/genomes): *Leuconostoc* (*Leu*.) *mesenteroides* ATCC 8293^T^ (GenBank accession no. CP000414), *Leu*. *lactis* DMLL10 (GenBank accession no. CP116456), *Bacillus* (*B*.) *subtilis* NCIB 3610^T^ (GenBank accession no. CP020102), *B*. *velezensis* DMB06 (GenBank accession no. CP083763), *B*. *siamensis* B28 (GenBank accession no. CP066219), *B*. *amyloliquefaciens* DSM 7^T^ (GenBank accession no. FN597644), *B*. *aerophilus* KJ82 (GenBank accession no. CP091093), *B*. *paralicheniformis* 14DA11 (GenBank accession no. CP023168), *Klebsiella* (*K*.) *grimontii* SS141 (GenBank accession no. CP044527), and *Mammaliicoccus* (*M*.) *sciuri* NCTC12103^T^ (GenBank accession no. LS483305) Amino acid biosynthetic pathways were predicted using Rapid Annotations using the Subsystems Technology (RAST) server for SEED-based automated annotation [17], then confirmed using the iPath (ver. 3) module [18] and CLgenomics ver. 1.55 software.

### Statistical analysis

Principal component analysis (PCA) was performed on the content of amino acids detected in both of the kimchi samples to visually differentiate between the bacterial communities of each kimchi sample during fermentation, using SPSS software v.27 (SPSS Inc., Chicago, IL, USA).

## Results and discussion

### Effects of *jogi* addition on color value

The effect of *jogi* addition on the color of kimchi was confirmed. In the case of BK without *jogi*, the L, *a*, and *b* values were 37.50 ± 0.22, 15.31 ± 0.18, and 22.78 ± 0.24, respectively. In the case of JBK with *jogi*, the L, *a*, and *b* values were 38.77 ± 0.17, 16.21 ± 0.10, and 22.80 ± 0.19, respectively, showing similar results to BK (Table 1). During the fermentation, L and *b* values decreased slightly, while in the case of BK, the *a* value increased slightly. However, in the case of JBK, the L, *a*, and *b* values all increased. These results assumed that the increase in L, *a*, and *b* values was due to *jogi* addition, but in the case of kimchi with Alaska Pollack, the *a* and *b* values decreased [19], and thus we suggest this to be a change by case, rather than a change due to seafood addition. The *a*/*b* ratio, which is the ratio of red to yellow, increased from 0.67 to 0.72 on the 20^th^ day of fermentation, and from 0.71 to 0.74. In the case of the overall change in color (Δ*E*), as the storage period increased, Δ*E* tended to increase, and all groups were in the numerical range 0 − 2.62, making the change difficult to distinguish with the naked eye. As the kimchi fermented, all samples, such as kimchi cabbage, seasoning, ingredients, and *jogi*, were mixed and aged, and the light yellow—white color of the *jogi* itself affected the L, *a*, and *b* values of kimchi, but the degree was weak, so there was no significant difference in color overall.

**Table 1:** Change of color of *Baechu—kimchi* and *Jogi—baechu—kimchi* during fermentation.

Fermentation period (days)		Sample	
		BK	JBK
L	0	37.50 ± 0.22^a,A^	38.77 ± 0.17^b,B^
	5	37.82 ± 0.06^a,A^	38.85 ± 0.78^b,B^
	10	37.85 ± 0.36^a,A^	38.77 ± 0.14^b,B^
	15	37.57 ± 0.33^a,A^	39.67 ± 0.30^c,B^
	20	37.44 ± 0.24^a,A^	39.40 ± 0.60^bc,B^
*a*	0	15.31 ± 0.18^a,A^	16.21 ± 0.10^bc,B^
	5	15.40 ± 0.29^a,A^	16.73 ± 0.15^cd,B^
	10	15.39 ± 0.06^a,A^	16.62 ± 0.33^cd,B^
	15	15.51 ± 0.04^ab,A^	17.09 ± 0.17^d,B^
	20	15.52 ± 0.03^ab,A^	18.11 ± 1.16^e,B^
*b*	0	22.78 ± 0.24^abc,A^	22.80 ± 0.19^abc,A^
	5	22.99 ± 0.07^bc,A^	22.82 ± 0.14^abc,A^
	10	22.47 ± 0.32^abc,A^	23.18 ± 0.09^bc,B^
	15	21.99 ± 0.15^ab,A^	23.48 ± 0.35^cd,B^
	20	21.62 ± 0.25^a,A^	24.49 ± 2.05^d,A^
*a/b*	0	0.67	0.71
	5	0.67	0.73
	10	0.68	0.72
	15	0.71	0.73
	20	0.72	0.74
Δ*E*	0	0	0
	5	0.40	0.53
	10	0.48	0.56
	15	0.82	1.43
	20	1.19	2.62

BK, *baechu—kimchi*; JBK, *jogi—baechu—kimchi*; L, lightness; *a*, redness; *b*, yellowness. This is the result of three repeated experiments.

^1) a, b, c, d, e^ Different superscripts in a column indicate significant differences at p<0.05 by Duncan’s multiple range test.

^2) A, B^ Different superscripts in a row indicate significant differences at p<0.05 by independent *t*-test.

### Effects of *jogi* addition on reducing sugar production

**Fig 1:**
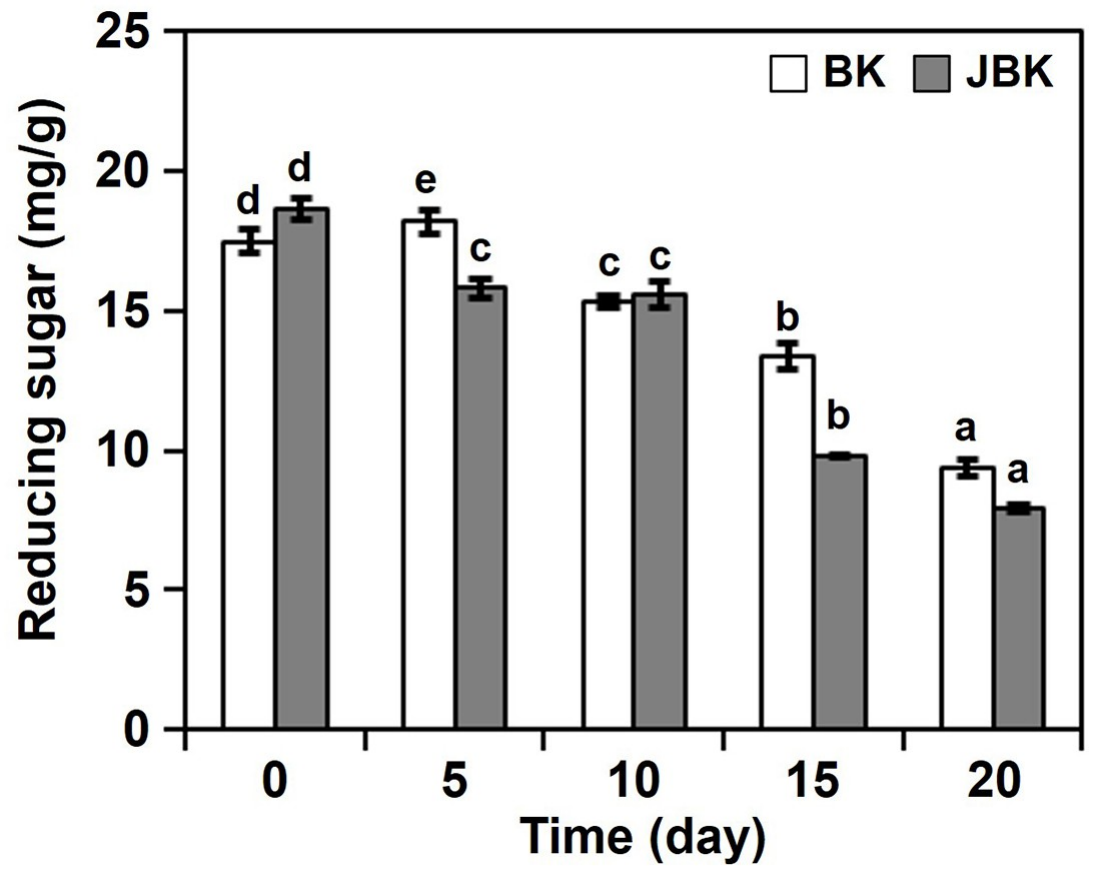
Effects of *jogi* addition of *baechu—kimchi* on reducing sugar production. BK, *baechu—kimchi*; JBK, *jogi—baechu—kimchi*; the following number indicates the fermentation time (in days). All values are presented as the mean ± SD of triplicate determination. Different letter indicates significant difference at *p*<0.05 using Duncan’s multiple range test.

In kimchi fermentation, reducing sugar is used as a carbon source for microorganisms, and is decomposed into organic acids and CO_2_; thus as fermentation proceeds, the content of reducing sugar decreases [20]. Since this decomposition of reducing sugar gives kimchi its unique taste and flavor, the degree of aging of kimchi, the degree of growth of microorganisms, and changes in flavor are also evaluated by examining the changes in reducing sugar [3]. To confirm the effect of *jogi* addition on the change in reducing sugar, the reducing sugar was analyzed. In the case of BK, on the 5^th^ day of fermentation, the reduction sugar content increased; but as fermentation progressed, it decreased (Fig 1). In addition, in the case of JBK, as fermentation progressed, it decreased, and the content of reducing sugar was less on 20^th^ day of fermentation, compared to the BK without *jogi*. As a result of the following literature survey to check if this was the result of *jogi* addition, the kimchi group with pollack added had a higher content of reducing sugar than the kimchi group without Pollack [19]. However, kimchi groups with octopus or squid were found to have lower levels of reducing sugar than kimchi groups without octopus or squid [7]. As a result of difficulty in affirming that by adding seafood, the reduction sugar has decreased or increased, these results are assumed to derive different results for each case. However, as fermentation progresses, reducing sugar clearly decreases.

### Effects of *jogi* addition on organic acid production

**Fig 2:**
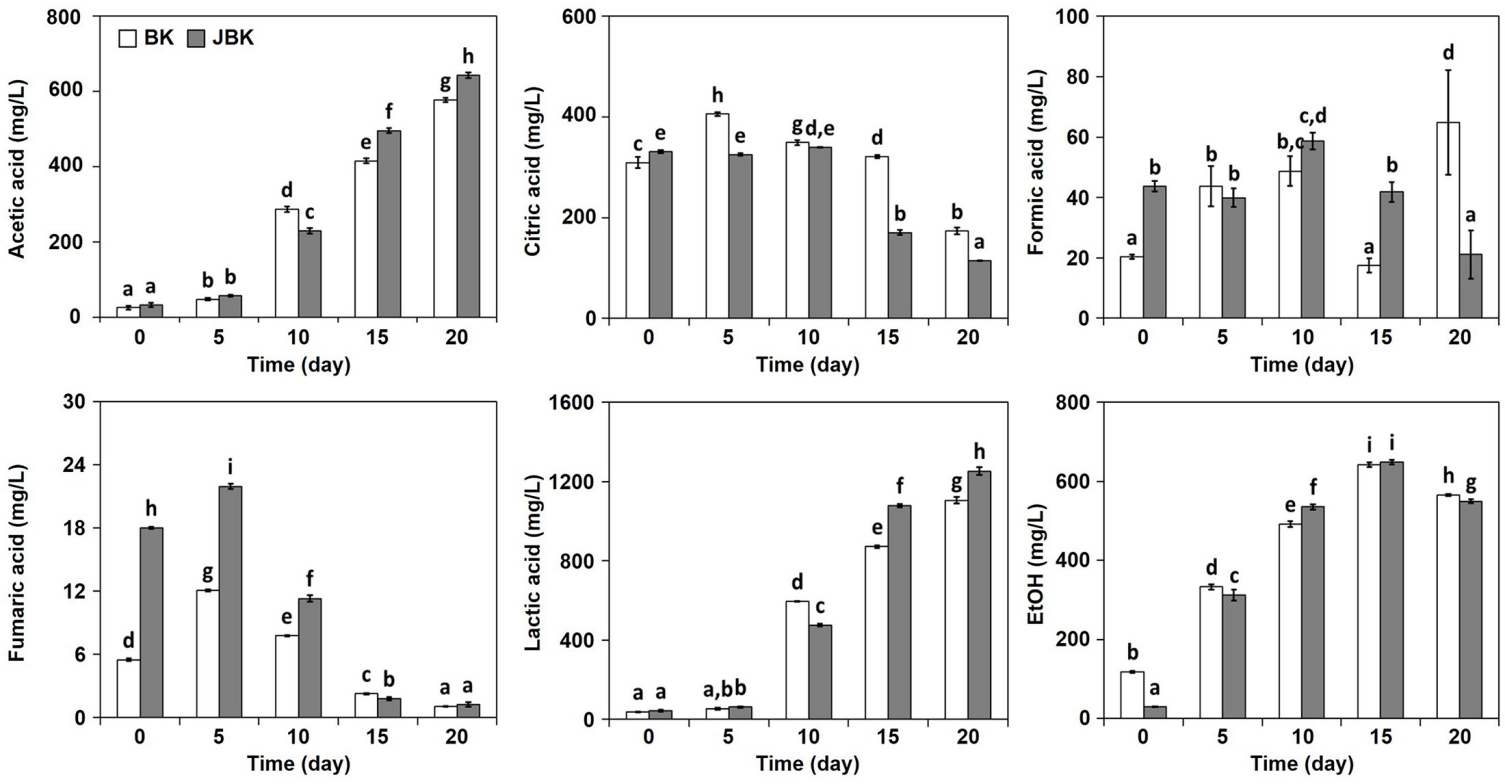
Effects of *jogi* addition of *baechu—kimchi* on organic acid production. BK, *baechu—kimchi*; JBK, *jogi—baechu—kimchi*; the following number indicates the fermentation time (in days). All values are presented as the mean ± SD of triplicate determination. Different letter indicates significant difference at *p*<0.05 using Duncan’s multiple range test.

Five organic acids and ethanol were identified in the two types of kimchi during fermentation (Fig 2). Citric acid was a major organic acid in the early stages of fermentation, but as fermentation progressed, it tended to gradually decrease. As fermentation progressed, acetic acid, ethanol, and lactic acid increased significantly, of which lactic acid increased the most. In comparison, the contents of formic acid and fumaric acid were not high during the fermentation period. Above all, formic acid showed no pattern of increase or decrease during the fermentation period. Accordingly, these results assumed that those two organic acids did not have a significant effect on kimchi fermentation. To determine the effect of *jogi* addition, we tried to confirm the change in organic acids between *baechu—kimchi* and *jogi*–*baechu—kimchi*, but it was confirmed that the effect on the production of organic acids was not significant.

Kimchi is a representative lactic acid fermented food, and the results of our experiments show that the amount of lactic acid increases over time, indicating that fermentation is proceeding well. What is interesting is that the sum of the acetic acid and ethanol contents is approximately same as the content of lactic acid. Ethanol, as well as lactic acid, were produced from the same molecule from one molecule of glucose via the hetero-fermentative pathway (or phosphoketolase pathway) under anaerobic condition [21]. On the other hand, the homo-fermentative pathway is known to produce lactic acid and acetic acid from glucose in the presence of oxygen [22]. Kimchi is not a perfectly controlled anaerobic fermentation. Therefore, based on the results, it was estimated that hetero-lactic fermentative LAB in contact with oxygen produces acetic acid, and in its absence, ethanol. To confirm this, the same kimchi used in this experiment was compared with the results of bacterial communities, which results are shown in previous studies [14]. It was found that the amount of bacterial communities was related to organic acids; Acetic acid, EtOH, and lactic acid increased from day 10 of fermentation (Fig 2), while *Leu*. *mesenteroides*, a heterolactic-fermentative LAB, was found to increase from day 10 and 5 of fermentation in the NGS results and culture-dependent results, respectively [14] (S1 Fig). These results suggest that acetic acid, EtOH, and lactic acid are produced by the influence of *Leu*. *mesenteroides*. However, in the culture-independent method (S1 Fig), *Lactobacillus sakei*, homo-lactic fermentative LAB, was more predominately detected than *Leu*. *mesenteroides*. *Lb*. *sakei* only produces two molecules of lactic acid from one molecule of glucose, so the lactic acid content should have been higher than the sum of acetic acid or ethanol [23]. Based on the content of organic acids (Fig 2), we assumed that during kimchi fermentation, *Leu*. *mesenteroides* could have influenced the production of organic acids, not *Lb*. *sakei*. To date, it is known that there is a difference in microbial aggregation according to culture-dependent and non-dependent methods [12, 14, 24], but considering the relationship with fermented products, we judged that it was more correlated with the results of culture-dependent methods.

### Effects of *jogi* addition on amino acid production

The effect of *jogi* addition on the microbial community was weak [14], and there was little effect on reducing sugar, organic acids, and color (Figs 1 and 2, Table 1). In general, kimchi with seafood, such as oysters, is delicious right after being made, and it is recommended to speed up consumption. Above all, it has been reported that the texture is related to the rich taste immediately after making kimchi [8, 19]. The amino acids that affect the umami flavor are glutamic acid and aspartic acid, and thus check the free amino acid containing two amino acids (Table 2). The increasing amino acids in fermentation for BK are alanine, arginine, leucine, lysine, phenylalanine, proline, tyrosine, and valine. The decreasing amino acids are aspartic acid, cysteine, glutamic acid, histidine, isoleucine, methionine, and threonine. In addition, JBK with *jogi* also shows similar results. Interestingly, the contents of glutamic acid and aspartic acid, which are rich taste components, in the early stages of fermentation, are higher in JBK than in BK (Table 2). Above all, on day 0, aspartic acid was three times higher than BK, while at day 15 of the fermentation, it was about 1.5 times higher. On day 0, glutamic acid was 1.2 times higher than BK, while on day 15 of fermentation, it tended to become similar. In addition, throughout the fermentation, alanine, arginine, glycine, isoleucine, leucine, lysine, methionine, phenylalanine, tyrosine, and valine showed higher levels than BK. In contrast, histidine, serine, and threonine were detected high in BK, and in the early stages of fermentation, showed higher values. This indicates that *jogi* addition is related to the content of amino acids, rather than color, reducing sugar, and organic acids. However, it is considered necessary through additional experiments to verify whether in this experiment, an effective amino acid is produced by the addition of seafood, such as premature fish, or an amino acid was produced only in this fermentation.

**Table 2:** Effects of *jogi* addition of *baechu—kimchi* on amino acid production. BK, *baechu—kimchi*; JBK, *jogi—baechu—kimchi*; the following number indicates the fermentation time (in days). All values are presented as the mean ± SD of duplicate determination. Different letter indicates significant difference at *p*<0.05 using Duncan’s multiple range test.

Amino acid (mg/L)	BK					JBK				
	0 day	5 day	10 day	15 day	20 day	0 day	5 day	10 day	15 day	20 day
Alanine	345.83±2.50^a,A^	341.98±35.55^a,A^	481.24±4.14^ab,A^	357.58±6.32^bc,A^	420.90±60.15^c,A^	349.37±8.21^ab,A^	488.14±12.31^cd,B^	435.27±62.47^c,A^	436.56±10.68^c,A^	548.04±20.61^d,A^
Arginine	886.19±37.86^a,A^	1126.67±215.66^b,A^	1200.84±84.47^bc,A^	1444.17±59.88^cd,A^	1625.77±182.51^de,A^	1198.70±26.50^bc,B^	1515.56±62.25^d,A^	1667.36±107.88^de,B^	1852.04±63.55^ef,B^	2016.12±1.34^f,A^
Aspartic acid	107.14±8.18^b.A^	110.65±20.64^b,A^	85.47±4.85^b,A^	95.38±5.07^a,A^	12.81±1.56^a,A^	280.22±10.92^e,B^	299.62±12.65^e,B^	223.48±19.58^d,B^	147.00±4.32^c,B^	31.94±0.78^a,B^
Cysteine	13.03±0.78^d,A^	12.03±0.42^cd,A^	9.72±1.05^b,A^	10.96±0.09^bcd,A^	6.48±1.11^a,A^	11.27±0.06^bcd,A^	12.12±0.40^cd,A^	10.25±1.60^bc,A^	6.58±0.24^a,B^	6.52±1.28^a,A^
Glutamic acid	360.96±15.79^c,A^	235.89±33.61^b,A^	191.81±2.94^a,A^	252.70±9.73^b,A^	250.10±30.24^b,A^	428.69±16.85^d,A^	341.93±14.95^c,A^	266.12±27.07^b,A^	260.75±9.77^b,A^	319.40±8.61^c,A^
Glycine	96.93±9.61^ab,A^	108.28±11.06^ab,A^	98.89±23.20^ab,A^	112.97±7.77^ab,A^	90.55±17.86^a,A^	105.58±1.09^ab,A^	111.61±2.91^ab,A^	113.71±9.22^ab,A^	113.28±1.33^ab,A^	123.59±2.50^b,A^
Histidine	1686.48±77.52^e,A^	1190.63±15.61^d,A^	1058.83±143.86^bcd,A^	1142.69±29.73^cd,A^	964.77±168.55^abc,A^	1081.83±17.64^bcd,B^	1210.46±40.89^d,A^	936.94±107.70^ab,A^	832.26±18.72^a,B^	856.54±2.04^a,A^
Isoleucine	72.65±4.50^a,A^	82.01±2.83^ab,A^	76.96±6.55^ab,A^	87.13±2.92^ab,A^	72.40±11.69^a,A^	87.35±1.88^ab,A^	86.76±3.36^ab,A^	90.41±12.53^b,A^	73.96±2.63^a,B^	87.05±1.63^ab,A^
Leucine	125.23±7.69^a,A^	158.89±3.76^bc,A^	149.85±14.90^abc,A^	175.97±3.03^cd,A^	145.66±23.33^ab,A^	158.64±3.03^bc,B^	158.55±6.06^bc,A^	175.82±22.52^cd,A^	163.22±5.19^bcd,A^	191.64±2.92^d,A^
Lysine	102.73±5.85^a,A^	145.67±22.10^bc,A^	136.89±4.42^b,A^	156.93±8.46^bcd,A^	137.05±19.18^b,A^	135.53±7.01^b,B^	158.88±6.91^bcd,A^	176.32±29.06^cd,A^	147.95±7.26^bc,A^	185.85±8.23^d,A^
Methionine	48.43±2.45^bc,A^	50.35±0.65^bc,A^	42.21±5.96^ab,A^	50.05±1.59^bc,A^	37.60±6.56^a,A^	74.09±1.05^e,B^	56.43±1.87^cd,B^	55.85±5.90^cd,A^	52.86±0.48^cd,A^	59.20±0.04^d,A^
Phenylalanine	100.23±6.05^a,A^	118.17±7.63^ab,A^	103.68±21.12^a,A^	122.66±12.12^ab,A^	105.66±17.95^a,A^	123.80±1.30^ab,B^	105.25±2.44^a,A^	115.94±4.80^ab,A^	122.94±2.07^ab,A^	135.91±3.41^b,A^
Proline	346.25±1.91^a,A^	463.46±42.33^b,A^	466.41±6.51^b,A^	493.97±11.46^b,A^	493.47±67.62^b,A^	289.35±6.07^a,B^	427.20±17.95^b,A^	501.82±62.68^b,A^	475.15±20.31^b,A^	447.65±19.13^b,A^
Serine	410.11±20.06^f,A^	323.08±4.67^e,A^	290.46±39.36^de,A^	320.90±7.47^e,A^	260.91±46.55^cd,A^	132.30±2.00^a,B^	138.33±5.00^a,B^	212.26±25.08^bc,B^	189.23±4.88^b,B^	237.73±1.02^bc,B^
Threonine	235.97±14.56^f,A^	234.33±8.76^ef,A^	189.54±31.14^cd,A^	199.65±9.40^cde,A^	135.90±24.51^a,A^	199.04±1.13^cde,A^	215.71±5.95^def,B^	171.40±17.40^bc,A^	128.49±2.80^a,A^	140.04±0.13^ab,A^
Tyrosine	83.38±4.91^a,A^	90.06±6.21^ab,A^	82.22±17.08^a,A^	95.60±10.09^ab,A^	101.66±16.48^ab,A^	92.65±1.49^ab,A^	82.38±0.93^a,A^	89.34±4.02^ab,A^	109.36±1.85^b,A^	112.00±9.68^b,A^
Valine	105.49±3.16^a,A^	118.22±4.90^ab,A^	113.17±8.38^ab,A^	133.35±1.32^bc,A^	115.08±18.72^ab,A^	117.76±2.97^ab,A^	124.04±5.06^abc,A^	133.11±18.53^bc,A^	124.59±3.80^abc,A^	146.38±3.13^c,A^

^1) a, b, c, d, e, f^ Different superscripts in a column indicate significant differences at p<0.05 by Duncan’s multiple range test.

^2) A, B^ Different superscripts in a row indicate significant differences at p<0.05 by independent *t*-test.

**Fig 3:**
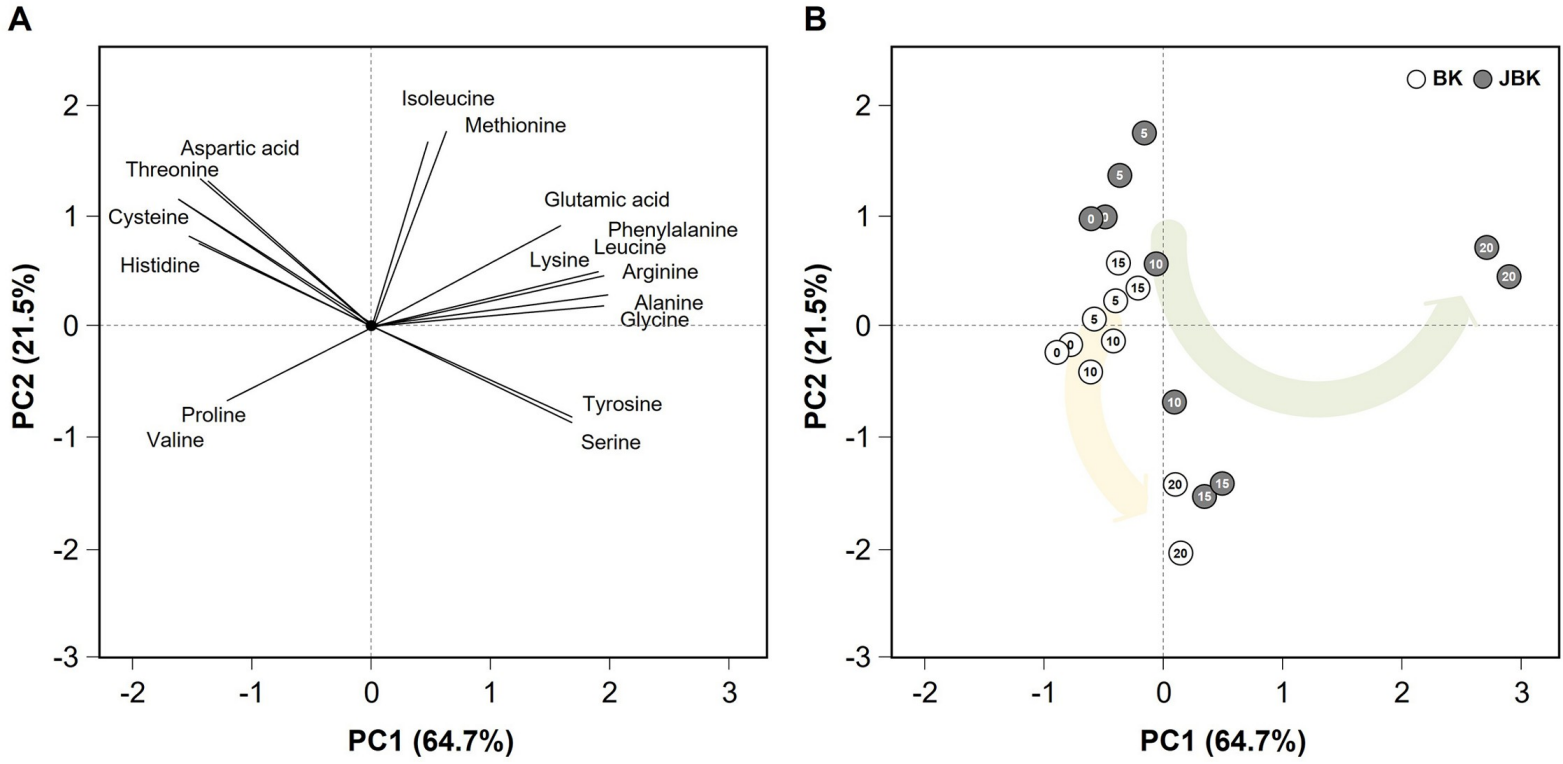
Principal component analysis based on amino acid production. BK, *baechu—kimchi*; JBK, *jogi—baechu—kimchi*; numbers beside symbols indicate fermentation time (in days). The curved arrow represents the path of amino acid change with fermentation time.

PCA analysis was performed through the SPSS statistical program using the measured amino acid content (Fig 3). In the PCA factor loading plot, aspartic acid, cysteine, histidine, threonine, proline, and valine were located in the negative direction based on PC1, while all other amino acids were located in the positive direction (Fig 3A). In the PCA score, each kimchi sample was divided by the fermented day (Fig 3B). On day 0 of fermentation, both JBK and BK were located in the negative direction, based on PC1 (Fig 3B). However, as fermentation progressed, both JBK and BK migrated in the positive direction, based on PC1. In particular, on day 20, JBK is in the positive direction, based on PC1; it is located in the first quadrant, which is the positive direction based on PC2; and the rich content of amino acids can be visually predicted from the location of this point. As can be seen in Table 2, these results show that on day 20, the content is high, except for the six amino acids: aspartic acid, cysteine, histidine, threonine, proline, and valine, out of 17 amino acids. It was visually confirmed that aspartic acid and glutamic acid of the JBK were also higher in content than in the BK. These results suggest that *jogi* addition has a greater impact on amino acid content than on color, reducing sugar, organic acid, and microbial community. In particular, the results clearly show that in the early stages of fermentation, there was a significant difference in the content of savory components, such as glutamic acid and aspartic acid.

### Correlation of amino acids and microbial communities

**Fig 4:**
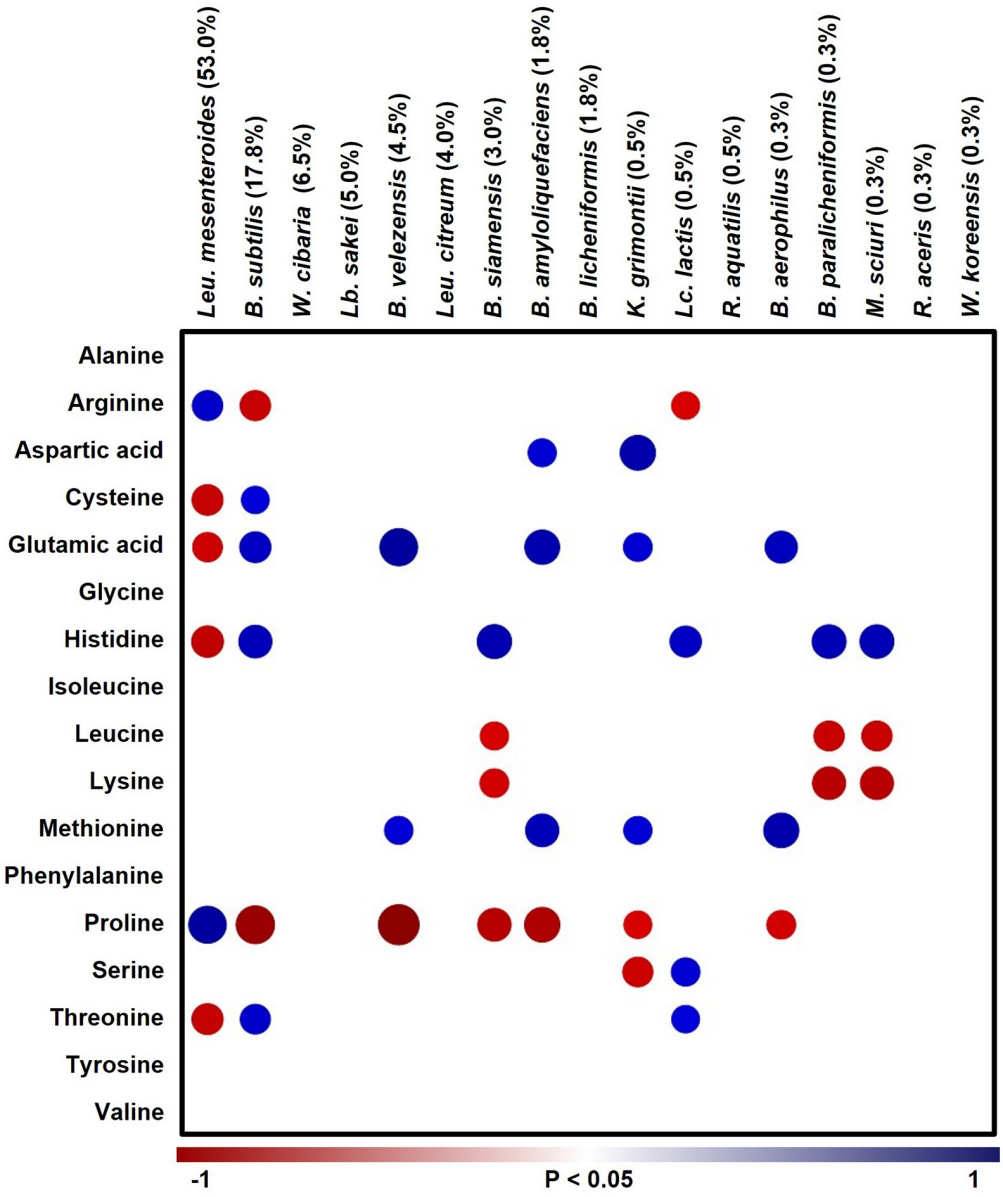
Correlation of amino acid and microbial community in kimchi. Only significant correlations (*p* < 0.05) are shown with a heat map; blue (positive) and red (negative), using Pearson correlation coefficient between the bacterial community at species level, and the amino acid contents of kimchi.

In summary, based on the results so far, the *jogi* addition affects the change in amino acid content from the beginning of fermentation. In addition, although there is no significant difference in the production of organic acids, it was judged that the production of bacterial community and organic acids derived by the culture-dependent method was more correlated than the results of the non-culture-dependent method. Therefore, the results were derived by analyzing the correlation between bacterial community analyzed by the culture-dependent method and the amino acids with a heat map (Fig 4). Most species have been found to exhibit some correlation with amino acids (Fig 4). However, 6 out of the 17 amino acids, namely alanine, glycine, isoleucine, phenylalanine, tyrosine and valine, were not correlated with microorganisms. *Leu*. *mesenteroides*, which accounted for 53% of the total microbial flora, formed a positive correlation between arginine and proline. *B*. *subtilis*, which accounted for 17.8%, showed a positive correlation with cysteine, glutamic acid, histidine, and threonine. Unusually, in the case of the genus *Weissella*, there was no correlation with amino acid production. The addition of *jogi* in the early fermentation period differed in the content of aspartic acid and glutamic acid, and the microorganisms that showed a positive correlation were *B*. *amyloliquefaciens*, *B*. *subtilis*, *B*. *velezensis*, *B*. *aerophilus*, and *K*. *grimontii*, all of which at the beginning of fermentation are species that dominate, and then decrease. Interestingly, the amino acids that are positively correlated with the genus *Bacillus* encoded the bio-synthetic genes for production of those amino acids via *Bacillus* genome analysis (S2 Fig) [25], whereas LAB, including *Leu*. *mesenteroides*, did not encode the synthetic genes (S2 Fig) [26]. This mainly suggests that during fermentation, amino acids were degraded by proteolytic enzymes from protein. *Bacillus* species and *K*. *grimontii*, which are positively correlated with a number of amino acids, possessed more protease genes, which degrade protein, than genomes of *Leu*. *mesenteroides* and *Leu*. *lactis* (S1 Table). Therefore, we assumed that the effect on the amino acid content is different, depending on the enzyme produced by the microorganism. Consequently, these results assumed that the amino acids contents were affected by bacteria biosynthesis, and that protease activity during fermentation could also have an effect.

It was expected that the addition of *jogi* to kimchi would increase the protein content and the content of bacteria that break down the protein, but contrary to our expectations, previous studies did not show much difference in microbial flora from the control groups without *jogi* addition [14]. In the current experiments, the results of confirming changes in physical and chemical components through *jogi* addition did not confirm dramatic changes in color, reducing sugar, and organic acid components (Figs 1 and 2, Table 1). However, in the case of amino acids, at the beginning of fermentation, aspartic acid and glutamic acid related to umami ingredients showed a significant difference in the *jogi* addition group, while on 20^th^ day of fermentation, most amino acids, including these two amino acids, were higher than in the control group (Table 2). Although the paper could not confirm that in the early stages of fermentation, the addition of seafood made excellent nutritional or sensual differences, word of mouth or universally known content recommends the consumption of seafood-added kimchi as quickly as possible, as kimchi immediately after manufacture is delicious. This general knowledge is probably understood to be due to the significant difference in taste when fish is added at the beginning of the fermentation. It is not just the difference in taste that encourages consumption within a short period of time, but it may also have included concerns that degrading enzymes from fish cause rapid fermentation, and shorten the period of shelf life. The results of this experiment can be meaningful, in so far as it is a scientifically proven fact that immediately after the preparation of kimchi, the *jogi* addition differs in the composition of the umami. However, these results are based on homemade kimchi and experiments conducted within 20 days after production. Therefore, it is suggested necessary to conduct experiments in the future to explore variations in factors such as the amount of *jogi*, extended fermentation periods, and fermentation temperatures.

## Supporting information

S1 Fig Relative abundance of the four most prevalent bacterial species in the microbiome of *baechu—kimchi* and *jogi—baechu—kimchi*, analyzed by (A) Culture-independent, and (B) Culture-dependent methods. This analysis is based on the results of a previously published paper [14].
(DOCX)

S2 Fig Predicted amino acid bio-synthetic pathways based on the genome of the 10 species. The names of enzyme-encoding genes and amino acids are depicted in green and white on orange, respectively. Black arrows correspond to potential enzymatic reactions catalyzed by gene products. When a strain possesses a gene, the appropriate color for the strain is shown in the box next to the gene name.
(DOCX)

S1 Table Putative protease genes identified in the genome of the 10 selected strains. The gene is denoted by the locus number of the genome. The Enzyme Commission (EC) number is a numerical classification scheme for enzymes, based on the chemical reactions they catalyze. Abbreviation: -, not identified or determined.
(XLSX)
