# Supplementary material for: The addition of jogi, Micropogonias undulates, affects amino acid content in kimchi fermentation
Source: PLoS One. 2024 Apr 4;19(4):e0300249. doi: 10.1371/journal.pone.0300249 (PMC10994411; doi:10.1371/journal.pone.0300249)

**S1 Fig. Relative abundance of the four most prevalent bacterial species in the microbiome of *baechu–kimchi* and *jogi–baechu–kimchi*, analyzed by (A) Culture-independent, and (B) Culture-dependent methods.** This analysis is based on the results of a previously published paper [14].


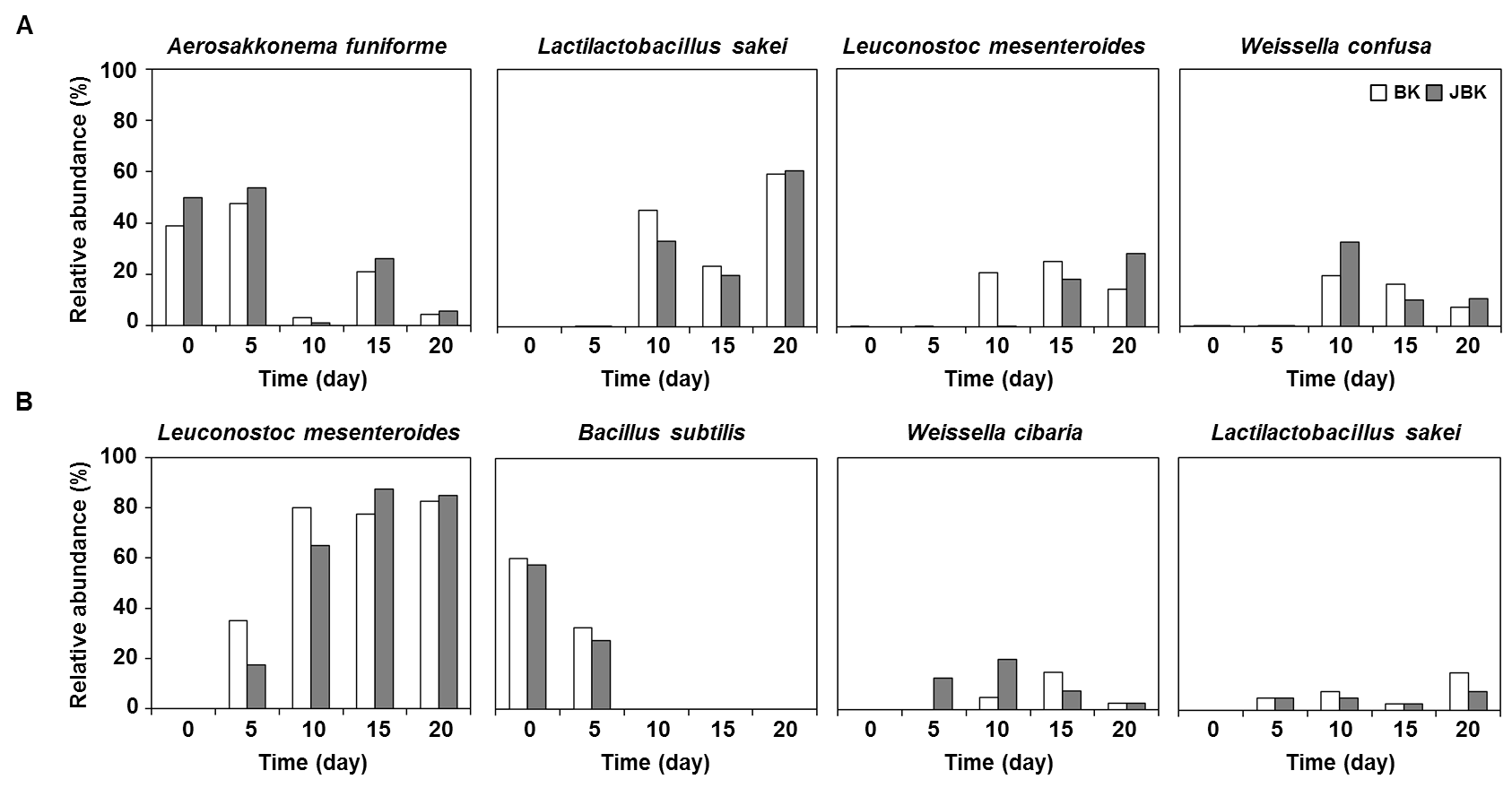

Supplement: S1 Fig — This analysis is based on the results of a previously published paper [14]. (DOCX) [file pone.0300249.s001.docx]
