# Supplementary material for: The addition of jogi, Micropogonias undulates, affects amino acid content in kimchi fermentation
Source: PLoS One. 2024 Apr 4;19(4):e0300249. doi: 10.1371/journal.pone.0300249 (PMC10994411; doi:10.1371/journal.pone.0300249)
Supplement: S2 Fig — The names of enzyme-encoding genes and amino acids are depicted in green and white on orange, respectively. Black arrows correspond to potential enzymatic reactions catalyzed by gene products. When a strain possesses a gene, the appropriate color for the strain is shown in the box next to the gene name. (DOCX) [file pone.0300249.s002.docx]

**S2 Fig. Predicted amino acid biosynthetic pathways based on the genome of the 10 species.** The names of enzyme-encoding genes and amino acids are depicted in green and white on orange, respectively. Black arrows correspond to potential enzymatic reactions catalyzed by gene products. When a strain possesses a gene, the appropriate color for the strain is shown in the box next to the gene name.

**
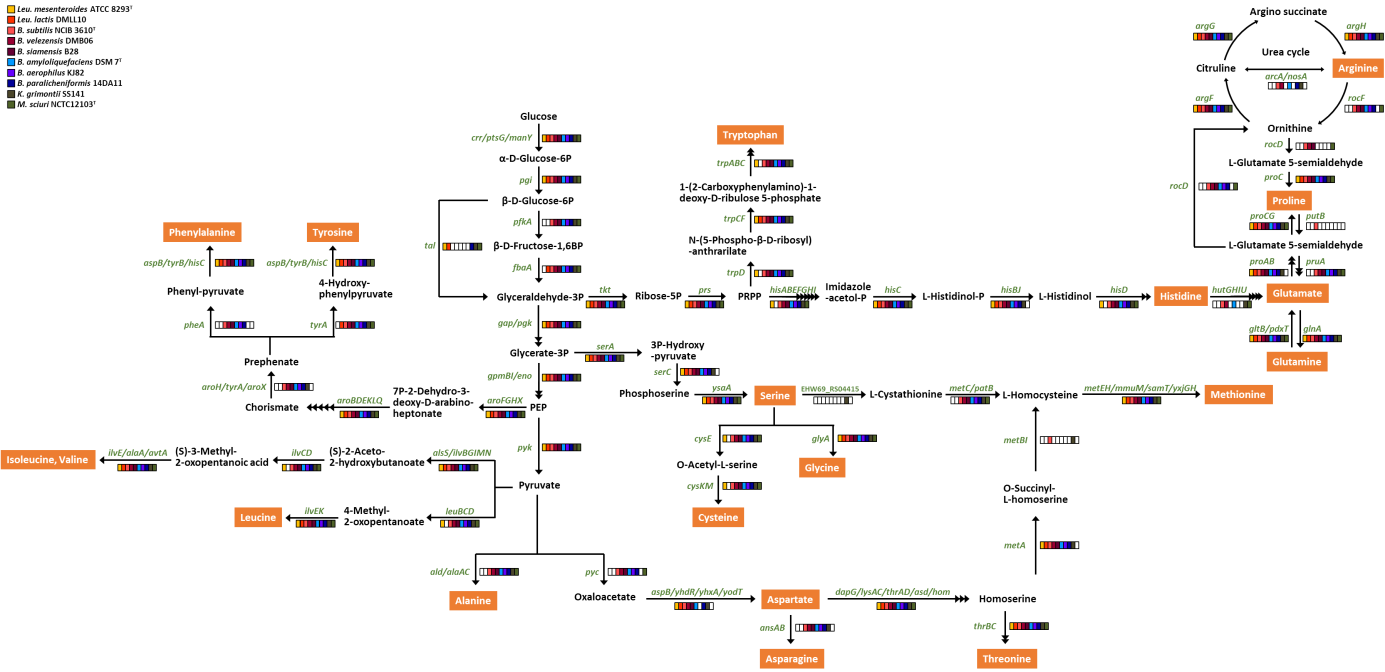
**
